# Association of serum chemokine (C–C motif) 21 and receptor (C–C motif) 7 with Hashimoto’s thyroiditis——a preliminary clinical investigation

**DOI:** 10.3389/fendo.2025.1682553

**Published:** 2025-10-29

**Authors:** Xiaoting Gui, Yuting Li, Yu Weng, Jiaxin Liao, Chunli Xiang, Xing Qi, Lizhang Xun, Feng Bai

**Affiliations:** ^1^ Department of Endocrinology, The Huai’an Hospital Affiliated to Xuzhou Medical University and The Second People’s Hospital of Huai’an, Xuzhou Medical University, Huai’an, China; ^2^ Department of Nephrology and Endocrinology, Ganzhou Hospital of Traditional Chinese Medicine, Ganzhou, Jiangxi, China; ^3^ Department of General Medicine, The Huai’an Hospital Affiliated to Xuzhou Medical University and The Second People’s Hospital of Huai’an, Xuzhou Medical University, Huai’an, China; ^4^ Department of Nephrology, Foshan Fosun Chancheng Hospital, Foshan, Guangdong, China; ^5^ Medical Examination Center, The Huai’an Hospital Affiliated to Xuzhou Medical University and The Second People’s Hospital of Huai’an, Xuzhou Medical University, Huai’an, China

**Keywords:** Hashimoto’s thyroiditis, thyroid autoantibody, chemokine C–C motif 21, chemokine C–C motif receptor 7, autoimmunity

## Abstract

**Objective:**

To investigate the associations of serum chemokine C–C motif 21 (CCL21) and chemokine C–C motif receptor 7 (CCR7) levels with the Hashimoto’s thyroiditis.

**Methods:**

We recruited 244 subjects (183 with Hashimoto’s thyroiditis and 61 healthy controls) from the Second People’s Hospital of Huai’an City. Patients with Hashimoto’s thyroiditis were divided into three groups according to the type of thyroid autoantibody: a. Double-positive group [elevated both thyroglobulin antibody (TgAb) and thyroid peroxidase antibody (TPOAb)]; b. TgAb group (elevated TgAb only); c. TPOAb group (elevated TPOAb only), they were further stratified according to thyroid function. We analyzed the between-group differences in the serum CCL21 and CCR7 levels which were quantified by enzyme-linked immunosorbent assay in all subjects.

**Results:**

Compared with the healthy control group, the Hashimoto’s thyroiditis group had significantly increased serum CCL21 [154.92 (147.01, 165.34) vs 123.74 (118.02,129.22), p<0.001] and CCR7 [19.15 (18.19, 19.71) vs 16.47 (15.88, 17.14), p<0.001], decreased free triiodothyronine, and increased thyroid stimulating hormone, TgAb, and TPOAb. Correlation analysis revealed that serum CCL21 levels were positively correlated with TgAb and TPOAb levels. Univariate binary logistic regression analysis showed that elevated serum CCL21 [OR = 2.944, 95% CI (1.464, 5.919), p=0.002] was significantly associated with Hashimoto’s thyroiditis. The results of receiver operating characteristic curve analysis indicated that CCL21 has value for the diagnosis of Hashimoto’s thyroiditis [AUC (95% CI)=0.998 (0.996-1.000), p<0.001]. Subgroup analysis suggested that serum CCL21 levels were significantly higher in the double-positive group than in the TgAb and TPOAb groups.

**Conclusion:**

Serum CCL21 is significantly elevated in patients with Hashimoto’s thyroiditis and correlates with both TgAb and TPOAb levels, suggesting that it is closely related to Hashimoto’s thyroiditis.

## 1 Introduction

Hashimoto’s thyroiditis is one of the most common autoimmune thyroid diseases (AITDs) and the leading cause of primary hypothyroidism in iodine-sufficient regions (1). Epidemiological data indicate that the prevalence of Hashimoto’s thyroiditis is greater among females than among males and increases with advancing age (2). The etiology and pathogenesis of Hashimoto’s thyroiditis have not been fully elucidated; however, evidence suggests that it is associated with genetic, environmental, and epigenetic factors (3), with autoimmunity being the recognized etiological factor (2). Lymphocytes play a crucial role in the development of Hashimoto’s thyroiditis.

Chemokines are a family of small cytokines with chemoattractant cellular activity that function by interacting with cell surface receptors. Chemokines are classified into four subfamilies on the basis of the number and location of N-terminal cysteine residues in different chemokines: XC, CC, CXC, and CX3C (4). The chemokine CCL21 is a CC chemokine that is produced primarily by stromal and lymphatic endothelial cells (5, 6). CCL21 exerts chemotactic effects on a wide range of cells, particularly lymphocytes, with a high degree of specificity and high efficiency (7, 8). CCR7, the primary binding receptor for CCL21, is a G protein-coupled receptor with seven alpha-helical transmembrane structures composed of hydrophobic amino acids. CCR7 is expressed predominantly in various immune cells as well as in tumor cells (9, 10).

CCL21 and CCR7 have been implicated in intrathyroidal lymphocyte infiltration, the formation of tertiary lymphoid organs in thyroid tissues, and the establishment of the thyroiditis microenvironment (11, 13). Martin et al. reported substantial lymphocyte infiltration in the thyroid glands of transgenic mice expressing CCL21 at the thyroglobulin promoter, whereas no thyroid lymphocyte infiltration was observed in CCL21 transgenic mice lacking CCR7 (12). A recent study demonstrated that the mesenchymal and immune cell components of Hashimoto’s thyroiditis drive thyroid lymphocyte infiltration and thyroid cell destruction. The findings disclosed that subpopulations of fibroblasts in the thyroid tissue of patients with Hashimoto’s thyroiditis expressed the chemokine CCL21 in significant quantities and that numerous CCL21+ fibroblasts were distributed in the T-cell area of the tertiary lymphoid organs of thyroid tissue. These findings indicated that CCL21+ fibroblasts likely contribute to lymphocyte recruitment and tertiary lymphoid organ formation in patients with Hashimoto’s thyroiditis (13).

Studies investigating the chemokine CCL21 and its receptor CCR7 in patients with Hashimoto’s thyroiditis are scarce in the literature. In the present study, we explored the associations of serum CCL21 and CCR7 levels with the risk of Hashimoto’s thyroiditis and evaluated differences in the expression of serum CCL21 and CCR7 in patients with different thyroid antibodies and thyroid function status.

## 2 Methods

### 2.1 Study design and registration

This was a prospective observational study. The study protocol was prospectively registered with the Chinese Clinical Trial Registry (registration number: ChiCTR2400091273) prior to the initiation of participant enrollment. Consecutive patients who met the inclusion and exclusion criteria were enrolled from October 2024 to January 2025 at Second People’s Hospital of Huai’an City. The present analysis constitutes a preliminary, cross-sectional examination of the baseline characteristics of the cohort. A total of 390 subjects were initially recruited. Based on the exclusion criteria, 146 participants were excluded for the following reasons: 31 lacked diffuse or heterogeneous echogenicity on thyroid ultrasound, 105 had incomplete data, 6 had a history of thyroid surgery, 1 was diagnosed with nodular goiter, 1 with subacute thyroiditis, and 2 tested positive for anti-thyroid hormone antibodies. Consequently, 244 participants were included in the final analysis, comprising 183 patients with Hashimoto’s thyroiditis and 61 healthy controls. The study protocol was conducted in accordance with the principles of the Declaration of Helsinki and received approval from the Ethics Committee of the Second People’s Hospital of Huai’an City (No. HEYLL202408).

### 2.2 Inclusion and exclusion criteria

The inclusion criteria for Hashimoto’s thyroiditis were as follows: 1) aged 18–75 years; 2) not restricted to a specific sex; and 3) thyroid ultrasound indicative of diffuse goiter with heterogeneous echogenicity and firm consistency, particularly with isthmus lobe enlargement, and positive serum antithyroglobulin antibody (TgAb) and/or antithyroid peroxidase antibody (TPOAb) (2, 14, 31). The exclusion criteria were as follows: 1) other thyroid diseases, such as Graves’ disease and subacute thyroiditis; 2) patients treated with glucocorticoids or levothyroxine therapy; 3) severe cardiopulmonary, hepatic, and renal insufficiency and malignant neoplasms; 4) severe stress, such as acute stroke and acute myocardial infarction, within the previous six months; 5) pregnancy and lactation; and 6) absence of essential laboratory data.

### 2.3 Research subgroups

Patients with Hashimoto’s thyroiditis were categorized on the basis of their thyroid autoantibody profiles as follows: double-positive group (elevated TgAb and TPOAb levels), TgAb group (elevated TgAb only, normal TPOAb), and TPOAb group (elevated TPOAb only, normal TgAb).

We subsequently classified patients with Hashimoto’s thyroiditis according to thyroid function into three subgroups: normal thyroid function, subclinical hypothyroidism, and clinical hypothyroidism. The diagnosis of subclinical hypothyroidism was established on the basis of thyroid-stimulating hormone (TSH) concentrations above the reference range and free thyroxine (FT4) and total thyroxine (TT4) levels within the normal range. Clinical hypothyroidism was diagnosed when TSH concentrations exceeded the reference range and when FT4 and TT4 concentrations fell below the reference range (32, 33).

Patients with Hashimoto’s thyroiditis were further stratified by body mass index (BMI) into subgroups to exploratively investigate the potential influence of obesity-related inflammatory status on the CCL21/CCR7 axis: normal group (BMI<24 kg/m2, n=87), overweight group (28 kg/m2<BMI ≤ 24 kg/m2, n=65), and obese group (BMI≥28 kg/m2, n=31).

### 2.4 Data collection

The demographic characteristics of all the subjects, including age, sex, systolic blood pressure (SBP), diastolic blood pressure (DBP), height, and weight, were obtained. Body mass index (BMI) was calculated using the following formula: weight (kg)/height (m)2.

### 2.5 Laboratory measurements

Fasting blood samples were collected from all the subjects after an 8-hour fast. The blood samples were then centrifuged at 3000 rpm for 20 min, and the resulting serum was stored at -80 °C until further analysis. Serum levels of TSH, free triiodothyronine (FT3), FT4, TgAb, and TPOAb were quantified using a Roche chemiluminescence assay. Total cholesterol (TC), triglyceride (TG), high-density lipoprotein cholesterol (HDL-C), and low-density lipoprotein cholesterol (LDL-C) levels were measured using an automated biochemical analyzer. The standard reference ranges provided by the laboratory center of the Second People’s Hospital of Huai’an City were as follows: TSH: 0.27~4.2 µIU/mL, FT3: 3.1~6.8 pmol/L, FT4: 12~22 pmol/L, TgAb<115 IU/ml, and TPOAb<34 IU/ml.

Upon sample collection, serum chemokine CCL21 and serum chemokine receptor CCR7 levels were quantified using identical model ELISA kits.

### 2.6 Thyroid ultrasound

All the subjects underwent thyroid ultrasound examination performed by an experienced sonographer using standard ultrasound equipment, and the report was diagnosed and reviewed by an attending physician. The subjects lay flat on the examination bed, the thyroid region was fully exposed, the probe was set to the appropriate frequency range, and multiple sections of the left lobe, right lobe, and isthmus of the thyroid were scanned to observe the morphology, size, presence or absence of nodules, texture, and blood flow, and the corresponding data were recorded.

### 2.7 Statistical analysis

Statistical analyses were performed and graphs were generated via SPSS 26.0 and GraphPad Prism 10. Normally distributed data are presented as the means ± standard deviations (SDs). Otherwise, the data are presented as medians (interquartile ranges). Categorical variables were analyzed using the chi-square test or Fisher’s exact test. We used to tests or Mann–Whitney U tests to compare continuous variables between the two groups. ANOVA and the Kruskal–Wallis H test were used to compare more than two groups, and Bonferroni’s method was used to correct for multiple comparisons. Correlations of serum CCL21 and CCR7 levels with other clinical variables were analyzed using Spearman’s correlation analysis. Binary logistic regression was used to preliminarily assess the relationship between CCL21 CCR7 levels and Hashimoto’s thyroiditis. The area under the curve (AUC) was calculated using the receiver operating characteristic (ROC) curve. All P values were two-tailed, and P < 0.05 was considered to indicate statistical significance.

## 3 Results

### 3.1 General information and clinical indicators for all the subjects

The study cohort comprised 244 subjects: 183 patients diagnosed with Hashimoto’s thyroiditis and 61 healthy control participants. Compared with the control group, the Hashimoto’s thyroiditis group presented elevated serum TSH, decreased serum FT3, and significantly increased serum TgAb and TPOAb levels (**Table 1**, P<0.05). Furthermore, patients with Hashimoto’s thyroiditis were more likely to have subclinical hypothyroidism. No statistically significant differences in sex, age, height, weight, BMI, SBP, DBP, FT4 level, or lipid level were detected between the two groups (**Table 1**, P>0.05).

**Table 1:** Characteristics of all the subjects.

Indicator	Control group (n = 61)	Hashimoto group (n = 183)	P value
Sex (female, %)	44 (72.1%)	137 (74.9%)	0.673
Age (years)	45.66 ± 11.07	45.42 ± 11.31	0.888
Height (m)	1.63 (1.59, 1.69)	1.62 (1.58,1.68)	0.440
Weight (kg)	63.30 (57.75, 73.25)	63.30 (56.90, 72.10)	0.672
BMI (kg/m)^2^	24.36 ± 3.14	24.46 ± 3.42	0.828
SBP (mmHg)	123.00 (113.50, 138.50)	123.00 (111.00, 138.00)	0.827
DBP (mmHg)	77.62 ± 10.61	76.04 ± 10.59	0.313
TSH (μIU/mL)	2.38 (1.65,2.89)	2.85 (1.91,4.48)	0.004*
FT3 (pmol/L)	5.16 (4.65, 5.73)	4.85 (4.42, 5.27)	0.003*
FT4 (pmol/L)	16.10 (15.30, 17.45)	16.10 (14.40, 17.80)	0.480
TgAb (IU/mL)	13.80 (13.00,15.10)	233.00 (103.00,371.00)	<0.001*
TPOAb (IU/mL)	10.10 (9.00, 13.05)	115.00 (25.00,315.00)	<0.001*
Hypo (n, %)	0 (0%)	7 (3.83%)	0.197
SCH (n, %)	0 (0%)	44 (24.04%)	<0.001*
TC (mmol/L)	4.79 (4.35,5.62)	4.75 (4.25, 5.39)	0.454
TG (mmol/L)	1.31 (0.98,1.82)	1.18 (0.90,1.76)	0.342
HDL-C (mmol/L)	1.31 (1.15,1.60)	1.36 (1.15,1.62)	0.467
LDL-C (mmol/L)	2.99 (2.57, 3.39)	2.75 (2.32, 3.40)	0.125
CCL21 (ng/L)	123.74(118.02,129.22)	154.92(147.01, 165.34)	<0.001*
CCR7 (pg/mL)	16.47 (15.88, 17.14)	19.15 (18.19, 19.71)	<0.001*

BMI, Body Mass Index; SBP, Systolic Blood Pressure; DBP, Diastolic Blood Pressure; Hypo, Hypothyroidism; SCH, Subclinical Hypothyroidism; TSH, Thyroid Stimulating Hormone; FT3, Free Triiodothyronine; FT4, Free Thyroxine; TgAb, Thyroglobulin Antibody; TPOAb, Thyroid Peroxidase Antibody; TC, Total Cholesterol; TG, Triglyceride; HDL-C, High-Density Lipoprotein Cholesterol; LDL-C, Low-Density Lipoprotein Cholesterol; CCL21, Chemokine C–C Motif 21; CCR7, Chemokine C–C Motif Receptor 7. *P < 0.05 was considered to indicate statistical significance.

### 3.2 Expression of serum CCL21 and CCR7

**Figure 1:**
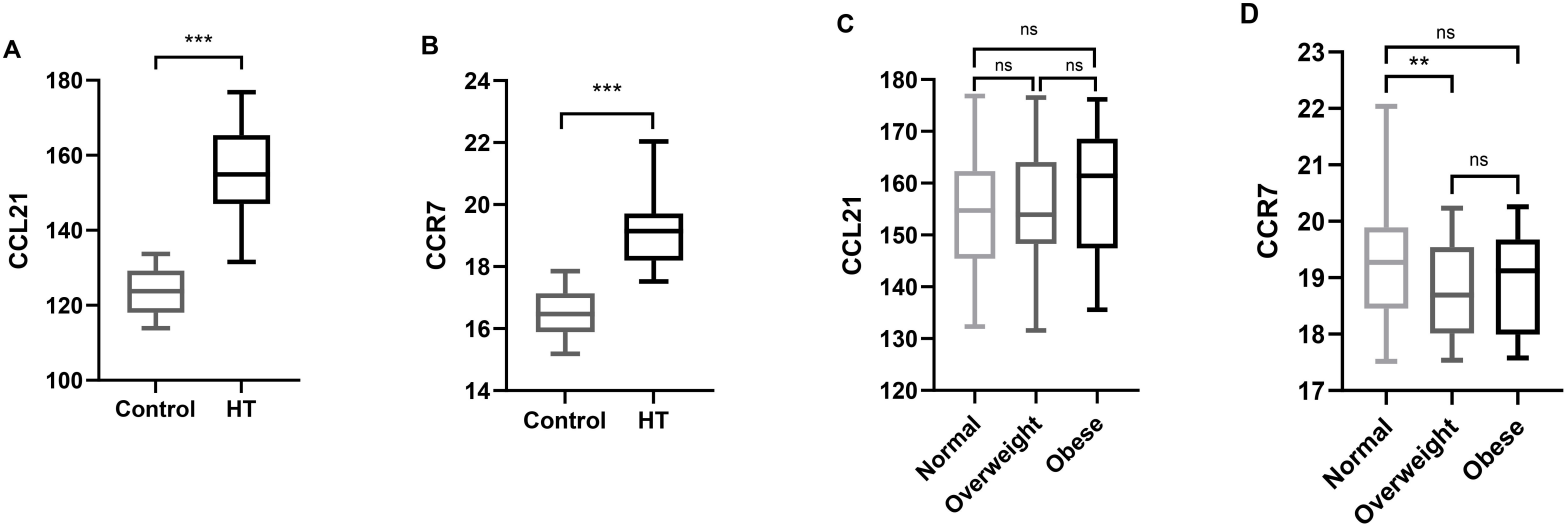
Serum CCL21 and CCR7 levels in the different groups. **(A)** Comparison of serum CCL21 levels between the control and Hashimoto groups. **(B)** Comparison of the serum CCR7 levels between the control and Hashimoto groups. **(C)** Comparison of serum CCL21 levels among normal weight, overweight, and obese Hashimoto patients. **(D)** Comparison of serum CCR7 levels among normal weight, overweight, and obese Hashimoto patients. Boxes indicate median and interquartile spacing. CCL21, Chemokine C–C Motif 21; CCR7, Chemokine C–C Motif Receptor 7; **P < 0.01, ***P < 0.001.

Serum CCL21 and CCR7 levels were measured in all the subjects and were found to be significantly elevated in the Hashimoto group compared with those in the control group (**Table 1**, **Figures 1A, B**, P < 0.001). The analysis showed no significant differences in CCL21 levels among normal, overweight, and obese patients. Serum CCR7 levels were significantly lower in overweight patients than in the normal BMI patients (P = 0.003), whereas no significant difference was observed between the other groups (**Figures 1C, D**). The serum CCL21 and CCR7 levels did not differ significantly according to sex or age in Hashimoto patients (data not shown).

### 3.3 The relationship between serum CCL21 and CCR7 with Hashimoto’s thyroiditis

Spearman correlation analysis suggested that serum CCL21 levels were positively correlated with DBP, TgAb, and TPOAb, serum CCR7 levels were negatively correlated with BMI and SBP (**Table 2**, P < 0.05). However our study revealed no significant correlation between serum CCL21 and CCR7 levels in patients with Hashimoto’s thyroiditis (**Table 2**, P > 0.05).

**Table 2:** Significant results of bivariate Spearman correlation analysis in the Hashimoto population.

Indicator	CCL21(ng/L)		Indicator	CCR7 (pg/mL)	
	r	p		r	p
Height (m)	0.031	0.678	Height (m)	0.022	0.771
Weight (kg)	0.093	0.209	Weight (kg)	-0.130	0.080
BMI (kg/m)^2^	0.110	0.140	BMI (kg/m)^2^	-0.201	0.006*
SBP (mmHg)	0.084	0.260	SBP (mmHg)	-0.159	0.032*
DBP (mmHg)	0.155	0.036*	DBP (mmHg)	-0.142	0.055
TSH (μIU/mL)	0.116	0.119	TSH (μIU/mL)	-0.110	0.137
FT3 (pmol/L)	0.022	0.764	FT3 (pmol/L)	-0.034	0.647
FT4 (pmol/L)	-0.026	0.727	FT4 (pmol/L)	-0.029	0.695
TgAb (IU/mL)	0.374	<0.001*	TgAb (IU/mL)	-0.084	0.257
TPOAb (IU/mL)	0.380	<0.001*	TPOAb (IU/mL)	0.051	0.496
TC (mmol/L)	-0.089	0.232	TC (mmol/L)	0.049	0.507
TG (mmol/L)	0.036	0.629	TG (mmol/L)	-0.006	0.938
HDL-C (mmol/L)	-0.097	0.190	HDL-C (mmol/L)	0.101	0.174
LDL-C (mmol/L)	-0.086	0.245	LDL-C (mmol/L)	0.006	0.931
CCR7 (pg/mL)	-0.019	0.794	CCL21(ng/L)	-0.019	0.794

BMI, Body Mass Index; SBP, Systolic Blood Pressure; DBP, Diastolic Blood Pressure; TgAb, Thyroglobulin Antibody; TPOAb, Thyroid Peroxidase Antibody; CCL21, Chemokine C–C Motif 21; CCR7, Chemokine C–C Motif Receptor 7. P < 0.05 was considered to indicate statistical significance. *p < 0.05, indicating statistical significance.

**Figure 2:**
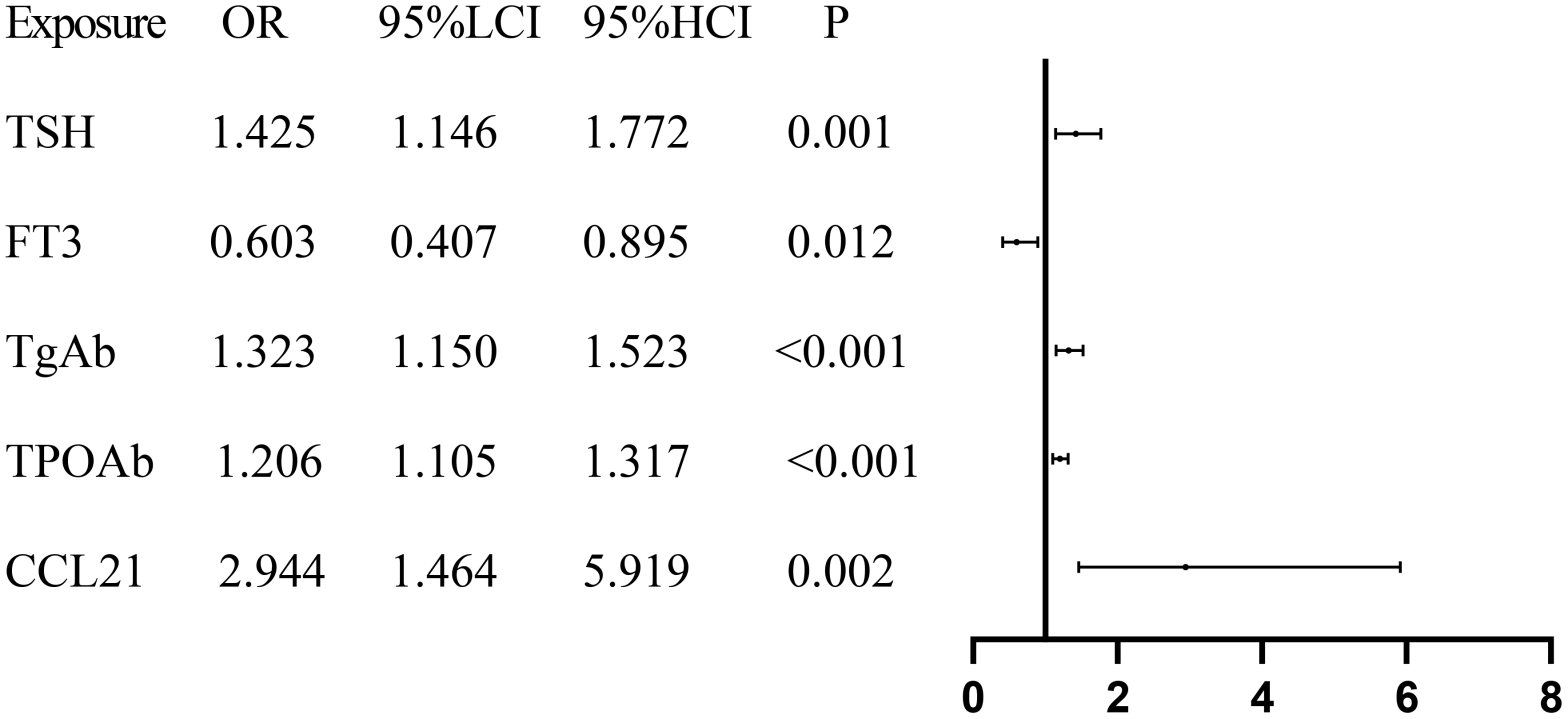
Forest plot presenting the results of regression analysis for Hashimoto’s thyroiditis. TSH, Thyroid Stimulating Hormone; FT3, Free Triiodothyronine; TgAb, Thyroglobulin Antibody; TPOAb, Thyroid Peroxidase Antibody; CCL21, Chemokine C–C Motif 21.

To analyze the relationships of serum CCL21 and CCR7 levels with the risk of developing Hashimoto’s thyroiditis, binary logistic regression analysis was performed, with the control group used as the reference. Univariate results showed that TSH, FT3, TgAb, TPOAb, and CCL21 were significantly associated with the risk of developing Hashimoto’s thyroiditis (**Figure 2**, P < 0.05). The results of the multivariate analysis indicated that none of the five variables retained statistical significance (**Supplementary Table 3**, all P > 0.05).

**Figure 3:**
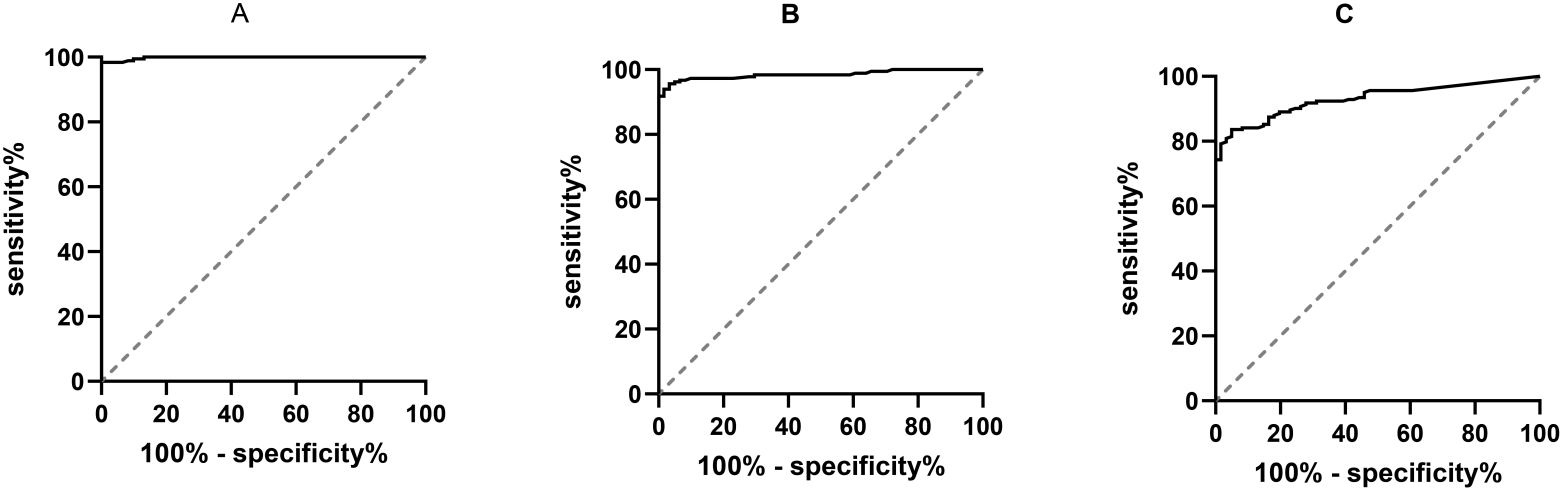
ROC curves of serum CCL21, TgAb, TPOAb, and Hashimoto’s thyroiditis. CCL21, Chemokine C–C Motif 21; TgAb, Thyroglobulin Antibody; TPOAb, Thyroid Peroxidase Antibody; ROC, Receiver Operating Characteristic; AUC, Area Under the Curve; **(A)** ROC curve results for CCL21; **(B)** ROC curve results for TgAb; **(C)** ROC curve results for TPOAb.

The ROC curve was plotted, and the results exhibited that the AUC for CCL21 was 0.998 (95% CI: 0.996-1.000, P < 0.001) (**Figure 3A**). The optimal cut-off value for CCL21 was 133.96, with a Youden index of 0.984, yielding a sensitivity of 0.984 and specificity of 1. The AUC for TgAb was 0.984 (95% CI: 0.971–0.998, P < 0.001) (**Figure 3B**). The optimal cut-off value for TgAb was determined to be 25.2, with a Youden index of 0.924, sensitivity of 0.940, and specificity of 0.984. The AUC for TPOAb was 0.930 (95% CI: 0.900–0.961, P < 0.001) (**Figure 3C**). The optimal cut-off value for TPOAb was determined to be 19.35, with a Youden index of 0.787, corresponding to a sensitivity of 0.836 and specificity of 0.951.

### 3.4 Subgroup analysis

Serum CCL21 levels were significantly greater in the double-positive group than in the TgAb and TPOAb groups (**Table 3**, P<0.001, P<0.001), and there was no significant difference in CCL21 levels between the TgAb and TPOAb groups. Serum CCR7 levels were not significantly different between the double-positive, TgAb, and TPOAb groups. The proportion of females in the TgAb group was significantly greater than that in the TPOAb group (P<0.05), the diastolic blood pressure in patients in the double-positive group was significantly greater than that in the TgAb group (P*=0*.036), and the systolic blood pressure in the double-positive group and the TPOAb group was significantly greater than that in the TgAb group (P = 0.017, P = 0.023). CCL21 showed significant positive correlations with both TgAb and TPOAb (**Table 2**, P<0.05). patients with Hashimoto’s thyroiditis were stratified by TgAb/TPOAb quartiles for further analysis. Compared to patients with antibody levels below the 25th percentile, patients with Hashimoto’s thyroiditis with antibody levels in P25-P50, P50-P75, and above P75 ranges showed significantly elevated serum CCL21 levels. However, CCL21 levels did not demonstrate a dose-dependent increase with progressively higher antibody tiers(**Supplementary Table 1**, P>0.05).

**Table 3:** Comparison of general information and data on different thyroid autoantibody types in Hashimoto’s thyroiditis patients.

Indicator	Double-positive group	TgAb group	TPOAb group	P value
Sex (female, %)	63 (74.1%)	44 (89.8%)	30 (61.2%)	0.005*
Age (years)	44.81 ± 10.85	44.82 ± 12.47	47.08 ± 10.96	0.489
Height (m)	1.63 (1.58, 1.68)	1.61 (1.58, 1.66)	1.62 (1.58,1.70)	0.317
Weight (kg)	65.2 (56.0, 72.2)	60.1 (56.4, 69.6)	64.9 (57.8, 73.1)	0.229
BMI (kg/m)^2^	24.70 ± 3.84	23.94 ± 3.04	24.57 ± 2.99	0.445
SBP (mmHg)	125.0 (113.5, 138.0)	115.0 (103.5, 136.0)	126.0 (116.5, 138.0)	0.031*
DBP (mmHg)	77.62 ± 10.85	72.86 ± 10.06	76.47 ± 10.17	0.040*
TSH (μIU/mL)	3.12 (2.20, 4.84)	2.32 (1.69,4.05)	2.59 (1.87,4.47)	0.056
FT3 (pmol/L)	4.96 (4.40, 5.41)	4.67 (4.36, 5.10)	4.98 (4.50,5.32)	0.063
FT4 (pmol/L)	15.70 (14.40, 17.55)	16.20 (14.50, 17.85)	16.50 (14.25, 18.05)	0.592
TgAb (IU/mL)	304.0 (208.0, 566.5)	262.0 (209.0, 380.5)	43.00 (26.15,79.45)	<0.001*
TPOAb (IU/mL)	211.0 (90.3, 417.0)	15.00 (11.05,21.25)	180.0 (102.0, 403.5)	<0.001*
Hypo (n, %)	4 (4.7%)	1 (2.0%)	2 (4.1%)	0.736
SCH (n, %)	24 (28.2%)	10 (20.4%)	10 (20.4%)	0.466
TC (mmol/L)	4.65 (4.12, 5.22)	4.82 (4.35,5.44)	4.93 (4.26, 5.67)	0.170
TG (mmol/L)	1.16 (0.92,1.64)	1.14 (0.93,1.72)	1.27 (0.79,2.11)	0.998
HDL-C (mmol/L)	1.36 (1.10, 1.62)	1.39 (1.21,1.62)	1.35 (1.15,1.66)	0.770
LDL-C (mmol/L)	2.64 (2.28, 3.12)	2.86 (2.46, 3.41)	2.86 (2.35.3.54)	0.151
CCL21 (ng/L)	166.01(160.24,170.50)	146.71(141.26,151.79)	148.26(139.12,151.21)	<0.001*
CCR7 (pg/mL)	19.06 (18.12, 19.63)	19.15 (18.31, 19.63)	19.26(18.21, 19.88)	0.524

BMI, Body Mass Index; SBP, Systolic Blood Pressure; DBP, Diastolic Blood Pressure; Hypo, Hypothyroidism; SCH, Subclinical Hypothyroidism; TSH, Thyroid Stimulating Hormone; FT3, Free Triiodothyronine; FT4, Free Thyroxine; TgAb, Thyroglobulin Antibody; TPOAb, Thyroid Peroxidase Antibody; TC, Total Cholesterol; TG, Triglyceride; HDL-C, High-Density Lipoprotein Cholesterol; LDL-C, Low-Density Lipoprotein Cholesterol; CCL21, Chemokine C–C Motif 21; CCR7, Chemokine C–C Motif Receptor 7. *P < 0.05 was considered to indicate statistical significance.

As shown in **Table 4**, exploratory analyses across the normal thyroid function, subclinical hypothyroidism, and clinical hypothyroidism groups revealed certain trends. we observed a tendency for TgAb, TPOAb, and serum CCL21 levels to gradually increase across the three groups, while serum CCR7 levels showed a tendency to decrease. However, likely due to the limited sample size, these trends did not reach statistical significance (P > 0.05).

**Table 4:** Comparison of thyroid function with serum CCL21, CCR7, and other clinical parameters in Hashimoto’s thyroiditis patients.

Indicator	Normal thyroid function (n=130)	Subclinical hypothyroidism (n=44)	Clinical hypothyroidism (n=7)	P value
TSH (μIU/mL)	2.26 (1.60,3.09)	5.37 (4.64, 7.36)	15.10 (6.95, 29.20)	<0.001
FT3 (pmol/L)	4.86 (4.43, 5.27)	4.92 (4.43, 5.32)	4.35 (4.23, 4.77)	0.146
FT4 (pmol/L)	16.40 (15.07, 18.02)	14.90 (13.9, 16.57)	10.20 (9.81, 11.50)	<0.001
TgAb (IU/mL)	216.00(89.70,352.25)	237.00(121.50,463.50)	417.00(103.00,1711.00)	0.236
TPOAb (IU/mL)	102.50(23.37,288.00)	153.00 (57.07,414.00)	236.00 (51.90,600.00)	0.300
CCL21 (ng/L)	153.53(146.94, 161.72)	157.27(145.93, 168.71)	162.37(148.29, 169.28)	0.522
CCR7 (pg/mL)	19.16 (18.28, 19.72)	18.96 (18.15, 19.72)	17.97 (17.74,20.03)	0.433
TC (mmol/L)	4.79 (4.29, 5.36)	4.72 (4.16, 5.33)	5.41 (4.46, 6.45)	0.191
TG (mmol/L)	1.19 (0.91,1.77)	1.13 (0.89,1.48)	1.39 (1.15, 2.51)	0.385
HDL-C (mmol/L)	1.35 (1.16,1.62)	1.41 (1.17, 1.62)	1.07 (0.97,1.69)	0.801
LDL-C (mmol/L)	2.76 (2.39, 3.40)	2.65 (2.29, 3.33)	3.49 (2.96, 4.36)	0.067

TSH, Thyroid Stimulating Hormone; FT3, Free Triiodothyronine; FT4, Free Thyroxine; TgAb, Thyroglobulin Antibody; TPOAb, Thyroid Peroxidase Antibody; CCL21, Chemokine C–C Motif 21; CCR7, Chemokine C–C Motif Receptor 7; TC, Total Cholesterol; TG, Triglyceride; HDL-C, High-Density Lipoprotein Cholesterol; LDL-C, Low-Density Lipoprotein Cholesterol. P < 0.05 was considered to indicate statistical significance.

According to thyroid ultrasound findings, patients comprised: Hashimoto’s thyroiditis with thyroid nodules, Hashimoto’s thyroiditis with thyroid cysts, and Hashimoto’s thyroiditis without nodules/cysts. No statistically significant differences were observed among the three groups in serum levels of TSH, FT3, FT4, TgAb, TPOAb, CCL21, or CCR7 (**Supplementary Table 2**, P > 0.05).

## 4 Discussion

Previous studies have demonstrated that CCL21 and its receptor, CCR7, are involved in numerous autoimmune and chronic inflammatory processes. Animal studies have shown that CCL21 expressed in AITD drives the recruitment of naive T and B cells to the thyroid (12). Qi et al. reported that the plasma chemokine CCL21 was strongly correlated with the activity and severity of Graves’ disease (15), that plasma CCL21 levels were significantly elevated in individuals diagnosed with primary Graves’ disease and TRAb-positive Graves’ disease and subsequently normalized in TRAb-negative Graves’ disease, and that CCL21 levels were positively correlated with TRAb levels. Our study showed that serum CCL21 levels were significantly elevated in the Hashimoto’s thyroiditis group compared with the control group and were positively correlated with TgAb and TPOAb. The binary logistic regression results indicated that the association between CCL21 and Hashimoto’s thyroiditis observed in the univariate analysis is not independent, but rather confounded by or mediated through its relationship with the established thyroid-specific antibodies (TgAb and TPOAb) and thyroid function parameters (TSH, FT3). In other words, CCL21 levels may be correlated with these core indicators of thyroid autoimmunity and dysfunction.

The CCR7/CCL19/CCL21 chemokine axis regulates the migration of a variety of an adaptive immune cells, particularly facilitating the homing of T and dendritic cells to lymphoid tissues (16, 17). Furthermore, CCR7 and CCL21 contribute to various adaptive immune functions, including secondary lymphoid organ formation (18), high-affinity antibody responses (19), and regulatory and memory T-cell functions (20, 21). CCL21, also referred to as secondary lymphoid tissue chemokine (SLC), was found to be significantly upregulated in the thyroid gland Hashimoto’s thyroiditis in a previous study (22). In addition, CCL21 was detected in endolymphoid follicles, and high endothelial microvessels (HEVs) were distributed around the endogenous centers of the thyroid gland, suggesting that this process occurs in thyroid and lymphoid follicles and is associated with the formation of secondary lymphoid follicles (23). Intrathyroidal lymphoid follicles respond to thyroid autoantigens and are associated with increased antibodies against thyroglobulin, thyroid peroxidase, and thyrotropin receptors (22). Our study showed a significant positive correlation between the serum chemokine CCL21 and thyroid autoantibodies in patients with Hashimoto’s thyroiditis. These studies suggest that CCL21 may play a crucial role in facilitating thyroid lymphoid neogenesis and driving thyroid autoimmunity. Our preliminary results indicated that serum CCL21 showed a significantly higher ROC value than TgAb and TPOAb for distinguishing patients with Hashimoto’s thyroiditis from healthy controls. While CCL21 showed remarkable discriminatory capacity in this cohort, we acknowledge that this finding may be influenced by the relatively selective nature of our study population. Therefore, the generalizability of these results needs to be verified in future multi-center, prospective studies.

CCR7 is expressed on various immune cells, and CCR7 interacts with CCL21 to regulate lymphocyte migration to secondary lymphoid organs. A previous study disclosed that (24) NOD background CCR7ko/ko mice presented a severe inflammatory response in thyroid tissue, which was markedly increased in appearance, and microscopic observation revealed infiltration of single nucleated cells, extensive destruction of the thyroid structure, and loss of thyroid follicular structure and fibrosis. NOD CCR7 ko/ko mice presented elevated TSH levels, decreased T4 levels, and a gradual increase in TgAb titers with age. These results suggest that CCR7 plays an important role in the development of autoimmune thyroiditis. Another study reported elevated levels of lymphotoxin α, lymphotoxin β, CCL21, CXCL12, CXCL13, and CCL22 in the thyroid glands of patients with autoimmune diseases and reported that the percentages of CXCR4+ T cells and CCR7+ B and T cells in the thyroid glands of patients with AITD were significantly lower than those in peripheral blood mononuclear cells (25). Our study indicated that serum CCL21 and CCR7 levels were significantly higher in patients with Hashimoto’s thyroiditis than in healthy individuals. However, the serum CCR7 level was not significantly correlated with the type of thyroid autoantibody or thyroid function in patients with Hashimoto’s thyroiditis, possibly because CCR7 is a cell membrane receptor, resulting in no significant difference being detected in the serum, in addition, limited by the small sample size. Further studies are needed to investigate the expression of CRR7 in lymphocyte subsets in patients with Hashimoto’s thyroiditis and the mechanisms and signaling pathways by which CCL21 mediates CCR7 to promote lymphocyte migration. Obesity is a chronic low-grade inflammatory state. We hypothesized that in patients with Hashimoto’s thyroiditis who are overweight or obese, this superimposed inflammation may modulate the CCL21/CCR7 axis—a signaling pathway implicated in immune cell migration and inflammatory responses. We found that serum CCR7 levels were significantly lower in overweight patients with Hashimoto’s thyroiditis than in those with normal weight. This inverse link suggests that an overweight state may be associated with the downregulation of CCR7 signaling within the HT context, potentially due to chronic metaflammation leading to altered immune regulation or compensatory receptor downregulation. However, no significant difference in serum CCR7 was observed between obese and normal-weight patients with Hashimoto’s thyroiditis, a finding that may be attributed to the limited sample size.

TgAb and TPOAb are characteristically present in patients with Hashimoto’s thyroiditis and are among the requirements for its diagnosis. In the pathogenesis of Hashimoto’s thyroiditis, TgAb is associated with the initial immune response, whereas TPOAb is linked to increased adaptive immunity (26). In a mouse model of spontaneous autoimmune thyroiditis, TgAb preceded the appearance of TPOAb (27). In human AITD, the majority of patients have been symptomatic for more than seven years when they present to a physician with a variety of clinical manifestations (28). Consequently, human patients with Hashimoto’s thyroiditis have a higher prevalence of TPOAb positivity than TgAb positivity (29). CCL21 mediates the migratory infiltration of immune cells, and the infiltration of immune cells into the thyroid gland produces autoantibodies against thyroid cell components. In this study, serum CCL21 levels were significantly positively correlated with TgAb and TPOAb levels. Furthermore, serum CCL21 levels were significantly higher in patients with Hashimoto’s thyroiditis who had elevated TgAb and TPOAb levels than in patients with Hashimoto’s thyroiditis who had elevated TgAb only or elevated TPOAb only. Previous studies have shown that the TPOAb titer is strongly correlated with the number of thyroid-infiltrating autoreactive lymphocytes (34) and the degree of ultrasound hypoechogenicity whereas TgAb was less sensitive. Our study showed that there were no significant differences in serum CCL21 and CCR7 levels between patients with Hashimoto’s thyroiditis with elevated levels of single antibodies, as well as the Hashimoto’s group with elevated single TgAb and the Hashimoto’s group with elevated single TPOAb had the same number of patients. This observation might be attributable to the limited sample size of Hashimoto’s thyroiditis patients enrolled in this study.

Patients with AITD often have abnormalities in thyroid function, and subclinical hypothyroidism is prevalent in patients with Hashimoto’s thyroiditis. Previous studies have reported that the prevalence of subclinical hypothyroidism varies between sexes, with values of approximately 3% in males and 5% in females, and that it is positively correlated with age (30). The typical pathology of Hashimoto’s thyroiditis involves lymphocyte infiltration of the thyroid tissue, with auto effector lymphocytes attacking and destroying the thyroid tissue, resulting in abnormalities in the secretion and regulation of thyroid hormones (31). Among 183 patients with Hashimoto’s thyroiditis in the present study, more than half had normal thyroid function, 44 patients had subclinical hypothyroidism, and seven patients developed clinical hypothyroidism. The results indicated that the serum CCL21 and CCR7 levels did not differ significantly between patients with Hashimoto’s thyroiditis with normal thyroid function and those with abnormal thyroid function. This observation might be attributed to the relatively small number of Hashimoto’s thyroiditis patients, particularly those with hypothyroidism, included in our study cohort.

Our study has several notable limitations that should be acknowledged. First, this was a single-center cross-sectional study with a small sample size, and certain confounding factors may have influenced the results. The reliance on hospital-based controls from health screenings may not be fully representative of the community, potentially affecting the observed associations as their exposure prevalence might differ from that of the general population. Particularly in the Hashimoto’s thyroiditis with abnormal thyroid function (n=51), substantially reduced our statistical power to detect clinically meaningful effects. In future studies, we plan to expand the sample size by including more hypothyroidism cases for further investigation. Longitudinal follow-up of the study population will be conducted to explore the relationship between baseline CCL21 levels and Hashimoto’s thyroiditis progression. Furthermore, our study did not compare CCL21 against other potential HT biomarkers; therefore, future research should aim to evaluate its relative contribution within a broader panel of biomarkers for a more comprehensive understanding of HT. Second, our results showed that serum CCL21 was significantly and positively correlated with TgAb and TPOAb, however, we could not determine a causal relationship between these factors. The pathogenesis of Hashimoto’s thyroiditis involves multiple factors and mechanisms. CCL21 drives lymphocyte infiltration to promote autoimmune responses, while the immune system mistakenly attacks the thyroid gland, resulting in the production of antibodies that subsequently activate complement and immune cells, thereby destroying thyroid cells and exacerbating the immune response. Third, we only examined serum CCL21 and CCR7 levels in the peripheral circulation, and the expression of CCL21 in thyroid tissue and that of CCR7 in peripheral blood lymphocytes remain unknown. Further investigation is warranted to elucidate the role of the CCL21/CCR7 axis in the pathogenesis of Hashimoto’s thyroiditis and evaluate its therapeutic potential as a novel treatment target.

In conclusion, the present study demonstrated that compared with those in healthy control participants, serum CCL21 levels were significantly elevated in patients with Hashimoto’s thyroiditis and were positively correlated with TgAb and TPOAb, which indicates that CCL21 was associated with the presence of Hashimoto’s thyroiditis. Serum CCR7 levels were elevated in patients with Hashimoto’s thyroiditis; however, serum CCR7 levels were not associated with serum CCL21 levels in patients with Hashimoto’s thyroiditis in this study. In the future, we will continue to further investigate the roles of CCL21 and CCR7 in Hashimoto’s thyroiditis.

## Data Availability

The original contributions presented in the study are included in the article/**Supplementary Material**. Further inquiries can be directed to the corresponding author.
