# Supplementary material for: Association of serum chemokine (C–C motif) 21 and receptor (C–C motif) 7 with Hashimoto’s thyroiditis——a preliminary clinical investigation
Source: Front Endocrinol (Lausanne). 2025 Oct 29;16:1682553. doi: 10.3389/fendo.2025.1682553 (PMC12604999; doi:10.3389/fendo.2025.1682553)
Supplement: Supplementary file 1 [file Table1.docx]

**Supplementary Table 1** Dynamic changes of serum CCL21, CCR7 and related variables across different antibody concentration ranges in Hashimoto's thyroiditis

| TgAb(IU/mL) | | | | | |
| --- | --- | --- | --- | --- | --- |
|  | <P25(n=48) | P25-50(n=43) | P50-75(n=46) | >P75(n=46) | P value |
| CCL21(ng/L) | 148.24  (139.03,151.24) | 158.58  (147.43,168.76) | 159.51  (144.33,168.39) | 159.58  (154.98,166.62) | <0.001 |
| CCR7(pg/mL) | 19.23  (18.17,19.88) | 19.21  (18.32,19.58) | 18.85  (18.00,19.63) | 19.04  (18.03,19.08) | 0.546 |
| TSH(uIU/mL) | 2.64  (1.89,4.56) | 2.80  (2.09,4.37) | 2.55  (1.75,4.41) | 3.13  (1.90,5.32) | 0.534 |
| FT3(pmol/L) | 4.96  (4.49,5.35) | 4.85  (4.43,5.27) | 4.86  (4.45,5.19) | 4.69  (4.22,5.34) | 0.553 |
| FT4(pmol/L) | 16.55  (14.35,18.07) | 16.20  (14.70,17.70) | 15.90  (14.40,17.85) | 15.60  (14.05,17.50) | 0.532 |
|  |  |  |  |  |  |
| TPOAb(IU/mL) | | | | | |
|  | <P25(n=47) | P25-50(n=44) | P50-75(n=46) | >P75(n=46) | P value |
| CCL21(ng/L) | 146.71  (141.78,150.63) | 158.18  (150.54,165.58) | 158.77  (149.72,167.21) | 161.05  (149.01,169.59) | <0.001 |
| CCR7(pg/mL) | 19.14  (18.21,19.59) | 19.15  (18.29,19.69) | 19.07  (18.00,19.75) | 19.15  (18.18,19.79) | 0.869 |
| TSH(uIU/mL) | 2.32  (1.68,4.37) | 2.59  (1.93,4.20) | 3.25  (2.31,4.49) | 3.15  (1.93,5.59) | 0.142 |
| FT3(pmol/L) | 4.67  (4.37,5.12) | 4.72  (4.28,5.13) | 5.08  (4.49,5.60) | 5.03  (4.65,5.29) | 0.017 |
| FT4(pmol/L) | 16.30  (14.70,17.90) | 16.15  (14.90,17.50) | 14.90  (13.87,17.92) | 16.40  (14.78,17.72) | 0.365 |

Abbreviations: TSH: Thyroid Stimulating Hormone; FT3: Free Triiodothyronine; FT4: Free Thyroxine; TgAb: Thyroglobulin Antibody; TPOAb: Thyroid Peroxidase Antibody; CCL21: Chemokine C–C Motif 21; CCR7: Chemokine C–C Motif Receptor 7. P < 0.05 was considered to indicate statistical significance.

**Supplementary Table 2** Variations in serum CCL21, CCR7 and related biomarkers across different thyroid ultrasound patterns in Hashimoto's thyroiditis

|  | Coexisting thyroid nodules(n=71) | Coexisting thyroid cysts(n=19) | Without nodules and cysts(n=101) | P value |
| --- | --- | --- | --- | --- |
| TSH(uIU/mL) | 2.57  (1.83,4.40) | 2.40  (1.18,3.03) | 3.09  (1.96,4.81) | 0.106 |
| FT3(pmol/L) | 4.86  (4.44,5.57) | 4.94  (4.29,5.27) | 4.85  (4.40,5.21) | 0.645 |
| FT4(pmol/L) | 16.00  (14.60,18.00) | 16.60  (15.60,17.90) | 16.00  (14.20,17.60) | 0.413 |
| TgAb(IU/mL) | 261.00  (98.60,361.00) | 168.00  (63.40,263.00) | 222.00  (112.00,415.50) | 0.234 |
| TPOAb(IU/mL) | 97.90  (21.90,305.00) | 166.00  (37.20,411.00) | 126.00  (26.05,306.00) | 0.640 |
| CCL21(ng/L) | 155.61  (144.58,165.64) | 154.95  (148.22,168.01) | 155.67  (147.42,164.47) | 0.613 |
| CCR7(pg/mL) | 19.04  (17.99,19.68) | 19.03  (18.27,19.68) | 19.17  (18.32,19,80) | 0.230 |

Abbreviations: TSH: Thyroid Stimulating Hormone; FT3: Free Triiodothyronine; FT4: Free Thyroxine; TgAb: Thyroglobulin Antibody; TPOAb: Thyroid Peroxidase Antibody; CCL21: Chemokine C–C Motif 21; CCR7: Chemokine C–C Motif Receptor 7. P < 0.05 was considered to indicate statistical significance.

**Supplementary Table 3** Multivariate logistic regression analysis of factors associated with Hashimoto's thyroiditis.

| Influencing factors | Coefficient of regression | P value | Exp(B) | 95%CI for Exp(B) | |
| --- | --- | --- | --- | --- | --- |
|  |  |  |  | LCI | HCI |
| TSH | 0.471 | 0.999 | 1.602 | 0.000 | - |
| FT3 | -3.133 | 0.998 | 0.044 | 0.000 | - |
| TgAb | 0.224 | 0.975 | 1.251 | 0.000 | 1659899.979 |
| TPOAb | 0.181 | 0.993 | 1.199 | 0.000 | 2.573E+18 |
| CCL21 | 1.202 | 0.991 | 3.328 | 0.000 | 1.648E+92 |

Abbreviations: TSH: Thyroid Stimulating Hormone; FT3: Free Triiodothyronine; TgAb: Thyroglobulin Antibody; TPOAb: Thyroid Peroxidase Antibody; CCL21: Chemokine C–C Motif 21; CCR7: Chemokine C–C Motif Receptor 7. P < 0.05 was considered to indicate statistical significance.
